# Voxel-wise correlations between cognition and cerebral blood flow using arterial spin-labeled perfusion MRI in patients with Alzheimer’s disease: a cross-sectional study

**DOI:** 10.1186/s12883-017-0870-x

**Published:** 2017-05-15

**Authors:** Tomohiro Kaneta, Omi Katsuse, Takamasa Hirano, Matsuyoshi Ogawa, Ayako Shihikura-Hino, Keisuke Yoshida, Toshinari Odawara, Yoshio Hirayasu, Tomio Inoue

**Affiliations:** 1 Department of Radiology, Yokohama City University, 3-9 Fukuura, Kanazawa-ku, Yokohama, 236-0004 Japan; 2 Department of Psychiatry, Yokohama City University, 3-9 Fukuura, Kanazawa-ku, Yokohama, 236-0004 Japan

## Abstract

**Background:**

To analyze voxel-wise correlation between cerebral blood flow (CBF) measured using ASL-MRI and cognition in patients with Alzheimer’s disease (AD).

**Methods:**

Forty-one patients diagnosed with AD or mild cognitive impairment due to AD were recruited for this study. CBF images were obtained using ASL-MRI (*n* = 41) with a post-labeling delay (PLD) of 1.5 and 2.5 s (PLD_1.5_ and PLD_2.5_, respectively) using a 3 T scanner, in addition to brain perfusion SPECT with *N*-isopropyl-4-[I-123]iodoamphetamine (*n* = 28). Voxel-based analyses were performed for ASL-MRI and SPECT using Mini-Mental State Examination (MMSE) scores as covariates. Differences in CBF between PLD_1.5_ and PLD_2.5_ were assessed using a paired t-test with SPM12.

**Results:**

Significant positive correlations were observed between MMSE scores and CBF at PLD_1.5_ in the right posterior cingulate cortex (PCC), and both temporo-parietal association cortexes. At PLD_2.5_, significant positive correlations were determined for MMSE scores and CBF in the superior parietal lobule and the right temporo-parietal association cortex. SPECT showed significant positive correlations in the PCC and both temporo-parietal association cortexes (right-side dominant). PLD_1.5_ showed significantly higher CBF than PLD_2.5_ in the proximal areas of vascular territories of the anterior, middle, and posterior cerebral arteries.

**Conclusions:**

Significant positive correlations in CBF, measured with both ASL-MRI and SPECT, with cognition were found in the PCC and temporo-parietal association cortexes. PLD_1.5_ and PLD_2.5_ showed similar correlations with cognition, although the CBF images had significant differences.

## Background

For the evaluation of cerebral blood flow (CBF) in patients with Alzheimer’s disease (AD), brain perfusion single photon emission computed tomography (SPECT) has been widely used for both visual and voxel-based analysis [1–6]. More recently, arterial spin labeling magnetic resonance imaging (ASL-MRI) has also been applied to evaluate CBF. This approach is a noninvasive technique that uses magnetically labeled water in arterial blood as an endogenous contrast medium [6–9]. Using this method to make comparisons between CBF in patients with various forms of dementia to demographically matched healthy controls has yielded significant findings. Both AD and mild cognitive impairment (MCI) have been associated with decreased CBF in the middle occipital areas, medial temporal lobe, and even more significantly in the parietal lobe [10]. Similar decreases in CBF have been reported in the posterior cingulate and precuneus, in addition to the frontal and parietal regions [11, 12]. ASL-MRI has also been successfully applied for the differential diagnosis of dementia [13, 14], and showed a high degree of concordance with FDG PET diagnoses [15, 16]. However, at present, few studies have reported voxel-wise correlations between CBF measured by ASL-MRI and cognition in patients with AD. The detection of regions with significant correlations between CBF and cognition may lend validity to the idea that regional CBF can serve as a biomarker of the neural changes underlying cognitive decline.

Among several ASL labeling approaches, pseudocontinuous ASL (PCASL) is now mainly used. This is a modification of continuous labeling in which a long train of short pulses is used to achieve flow-induced adiabatic inversion of arterial blood water, and is recommended for clinical imaging by the International Society for Magnetic Resonance in Medicine (ISMRM) study group on perfusion imaging [17]. Arterial water at the base of the brain is labeled by the PCASL pulses, and the brain is imaged after a fixed time interval. This is termed post-labeling delay (PLD) and is a key parameter for ASL imaging. Several values of PLD such as 1.5, 2.0, and 2.5 s have been used in the studies of AD [18–20]. Generally, PLD is fixed to one value, but using multiple PLDs seems interesting to evaluate the optimal PLD and the difference between varying PLDs. In addition, the influence of different PLDs on the results of voxel-wise analyses has not been evaluated.

In this study, we evaluated and compared the correlation between cognition and CBF measured by ASL-MRI with a PLD of 1.5 s and 2.5 s (PLD_1.5_ and PLD_2.5_, respectively) in patients with AD. We also compared the results with brain perfusion SPECT.

## Methods

### Subjects

Forty-one patients who underwent MRI with diagnosis of AD or mild cognitive impairment (MCI) due to AD between September 2015 and March 2016 were recruited. Patients with AD were included if they met the criteria for probable AD established by the National Institute of Neurological and Communicative Disorders and Stroke-Alzheimer Disease and Related Disorders Association (NINCDS/ADRDA) [21]. Patients were excluded from the study if they had a significant history of psychiatric or neurological disorders other than AD, including stroke, head injury, epilepsy, psychiatric disorders, alcohol abuse, or other serious medical conditions. All patients underwent MR scanning at 3.0 T and standard dementia screening, which included a medical history check, Mini-Mental State Examination (MMSE), neuropsychological testing, and MR imaging. Cognition was assessed using the MMSE, which evaluates general cognitive function, including orientation to time and place, attention and calculation, language, and memory [22]. Among these, 28 patients also underwent brain perfusion SPECT using *N*-isopropyl-4-[^123^I]iodoamphetamine (^123^I–IMP) within 1 month of MR scanning at our hospital. This tracer has been reported to be feasible for the quantitative evaluation of CBF in routine clinical practice [23]. Most of others underwent SPECT using other tracer or at another hospital, but we did not include their SPECT images for the analysis in this study. We used all available images of both ASL and SPECT obtained from all patients.

### MR imaging

Images were acquired using a 3.0 T MR system (Discovery750w, GE Medical Systems) and a 12-channel head coil.

Anatomic information was obtained from a sagittal three-dimensional (3D) T1-weighted sequence, (the parameters include: TR = 6.6 ms, TE = 2 ms, 14° flip angle, matrix = 256 × 256, 170 stions, voxel size = 1.0 × 0.9 × 0.9 mm^3^, FOV = 23 × 23 cm), acquisition time was 6 min 0 sec.

The ASL sequence consisted of a 3D, multi-delay PCASL, with a fast spin-echo acquisition with background suppression. The labeling plane was set at the base of the brain without the information of MR angiography. The imaging protocol of PLD_1.5_ was as follows: TR = 4641 ms, TE = 10.7 ms, locations = 36, FOV = 23 × 23 cm, voxel size = 2 × 2 × 4 mm^3^, PLD = 1.5 s, labeling duration = 1.5 s, number of excitations (NEX) = 1, acquisition time was 1 min 33 s. The imaging protocol of PLD_2.5_ was as follows: TR = 5336 ms, TE = 10.7 ms, PLD = 2.5 s, NEX = 2, all other parameters maintained the same, and the acquisition time was 2 min 51 s.

A two-compartment model with finite labeling duration was used for PCASL quantification. An approximately proton-density-weighted image was obtained by turning the labeling RF off. Calculation of flow was based on the following equation.

1 $$ \mathrm{f}=\frac{\lambda \left({S}_{ctrl}-{S}_{lbl}\right)\left(1-{e}^{-\frac{t_{sat}}{T_{1 g}}}\right)}{2\alpha {T}_{1 b}\left(1-{e}^{-\frac{\tau}{T_{1 b}}}\right){S}_{ref}}{e}^{\frac{w}{T_{1 b}}} $$

where *f* is the measured CBF; *S* is the signal from the control, label, or reference image as determined by the subscript, *S*_lbl_ is the label image, i.e. image obtained with unbalanced RF labeling that gives rise to perfusion weighting, *S*_ctrl_ is the control image, i.e. image obtained with balanced RF so that the arterial blood is not labeled, *S*_ref_ is the proton density image that is obtained with labeling RF turned off; *T*_1_ *b* is the T1 of blood; T_1_ *g* is the T1 of gray matter; α is the labeling efficiency; λ is the brain–blood partition coefficient; *t*_sat_ is the saturation time for proton density images (2 s); τ is the labeling duration (1.5 s); and ω is the post label delay. We used a gray matter T1 estimate of 1.2 s and an assumed blood T1 of 1.6 s [24]. The labeling efficiency was assumed to be 0.8 for the PCASL.

### Preprocessing and MR imaging data analysis

Data analyses were carried out by using Statistical parametric mapping 12 (SPM12) (http://www.fil.ion.ucl.ac.uk/spm/software/spm12/). Both 3D T1-weighted and 3D PCASL images were corrected for image distortion due to gradient non-linearity using ‘GradWarp’ [25]. Preprocessing of 3D T1-weighted images consisted of realignment, coregistration, and segmentation.

ASL images were linearly registered to the brain extracted from the 3D T1-weighted images. Mean whole-brain CBF values were calculated in the brain mask, converted to quantitative CBF maps in the unit of mL/100 g/min, spatially normalized to the Montreal Neurological Institute (MNI) space with a 2-mm isotropic resolution, and smoothed with an isotropic kernel of 6 mm. Complementary voxel-wise comparisons of 2 kinds of CBF maps were performed by SPM12 as well. Correlation results were statistically thresholded at *p* < 0.001 uncorrected. All results were shown in the MNI space. The locations with significant results were determined using the “Neuromorphometrics” function of SPM12.

### Brain perfusion SPECT

SPECT was performed by intravenous injection of 148 MBq ^123^I–IMP (Nihon Mediphysics, Hyogo, Japan) in participants seated at rest with their eyes open. A dual-head gamma camera with integrated thin-slice diagnostic CT (Symbia® T16, Siemens Healthcare, Molecular Imaging, Hoffman Estates, IL, USA) was used. The SPECT scans were acquired using low-medium-energy general-purpose collimation, a 128 × 128 matrix of 3.3-mm pixel size, and a total of 300 s/rotation in a continuous-rotation mode. Subsequent to the SPECT acquisition, a reduced-dose CT scan was acquired with 130 kV and 150 ref. mAs. The CT data were generated with a 3-mm slice thickness using a smooth reconstruction kernel (H08s, Siemens Healthcare) and a 2-mm slice thickness using a medium kernel (H31s medium sharp, Siemens Healthcare).

SPECT reconstruction was performed using filtered back projection using a Butterworth filter with cutoff = 0.35/cm and order 8. A uniform attenuation correction was performed using Chang’s method with μ = 0.11.

### Statistical analysis

Voxel-based analyses were performed for PLD_1.5_, PLD_2.5_ and brain perfusion SPECT using MMSE scores as covariates. Differences of CBF between PLD_1.5_ and PLD_2.5_ were assessed by paired t-test. The individual brain images were normalized by the global values. The *p* value threshold was 0.001 at the voxel level, and the regions with the extent under the expected voxels per cluster were omitted. The plots of the correlation between the voxel values and MMSE scores at the most significant area are provided. These were performed by SPM12.

## Results

### Patient demographics

Patient demographics, including age, sex, and MMSE scores, are summarized in in Table 1. There were no significant differences between the total population (*n* = 41) and the subpopulation that underwent SPECT (*n* = 28).

**Table 1:** Demographic and neuropsychological summary of subjects included in the current study ^a^

	Total	SPECT
Age, y	79.0 (6.4)	79.5 (6.6)
No. of patients	41	28
Gender, M/F	17/24	9/19
MMSE	23.0 (3.8)	23.0 (3.9)

^a^Data are presented as mean (SD).

### Voxel-wise correlations between CBF and cognition

**Fig. 1:**
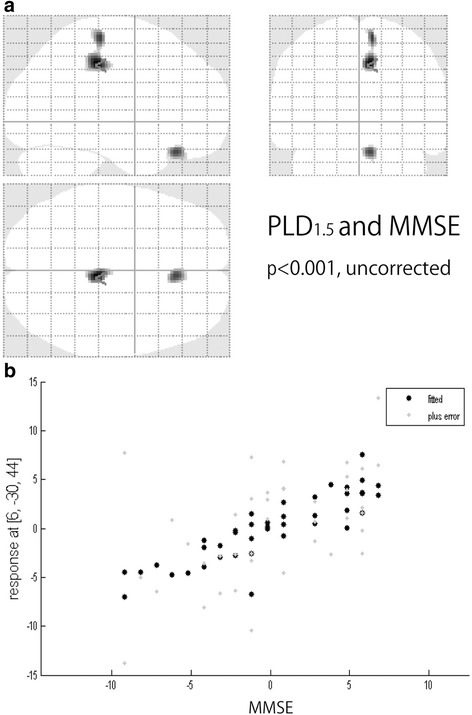
**a** Voxel-wise correlations between cognition measured with MMSE and CBF measured using PLD_1.5_ (*p* < 0.001, *T* = 3.32, uncorrected). **b** The plots of the correlation between the voxel values and MMSE scores at the most significant area (*right posterior cingulate cortex*)

**Fig. 2:**
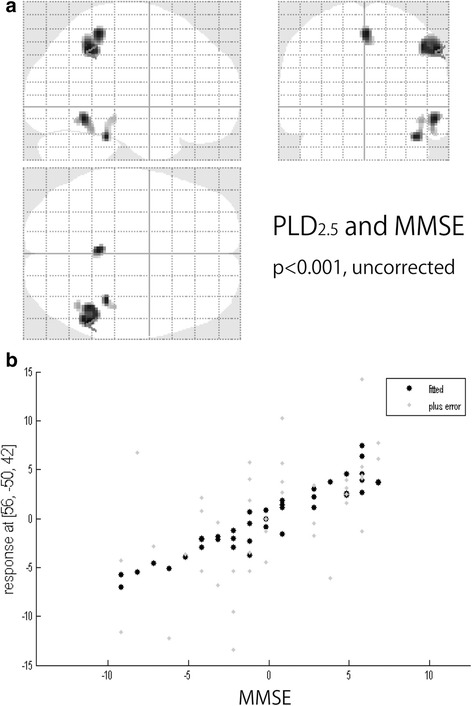
**a** Voxel-wise correlations between cognition measured with MMSE and CBF measured using PLD_2.5_ (*p* < 0.001, *T* = 3.32, uncorrected). **b** The plots of the correlation between the voxel values and MMSE scores at the most significant area (*right inferior parietal lobule*)

**Fig. 3:**
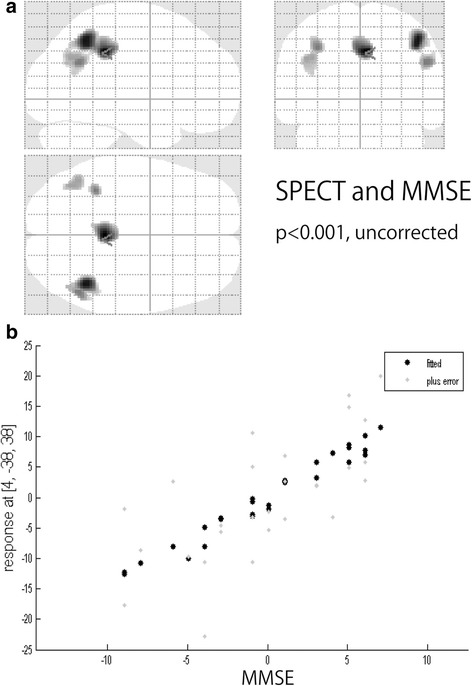
**a** Voxel-wise correlations between cognition measured with MMSE and CBF measured using brain perfusion SPECT (*p* < 0.001, *T* = 3.45, uncorrected). **b** The plots of the correlation between the voxel values and MMSE scores at the most significant area (*right posterior cingulate cortex*)

Using ASL-MRI, PLD_1.5_ scans revealed significant positive correlations with MMSE results in the right posterior cingulate cortex (PCC) and both temporo-parietal association cortexes, in addition to the right rectal gyrus (Fig 1a). PLD_2.5_ showed significant positive correlations in the superior parietal lobule but not PCC, and the right temporo-parietal association cortex only. Positive correlations for the right inferior temporal lobule and right fusiform gyrus were also significant (Fig 2a). Brain perfusion SPECT identified significant positive correlations in the PCC and both temporo-parietal association cortexes (right-side dominant), and the left fusiform gyrus (Fig 3a). The plots of the correlation between the voxel values and MMSE scores at the most significant area are shown in Figs. 1b, 2b, and 3b. All of them showed linear relationships. The expected voxels per cluster for PLD_1.5_, PLD_2.5_, and SPECT were 63.4, 40.8, and 60.9, respectively. Cluster-level statistics for all rendered clusters are summarized in Table 2.

**Table 2:** Summary of voxel-wise CBF correlations with MMSE

Modality	Anatomic label	x	y	z	t statistic	z score	Voxels
PLD_1.5_	R PCC	6	−30	44	4.65	4.11	209
	R gyrus rectus	6	30	−26	4.20	3.79	97
PLD_2.5_	R IPL	56	−50	−42	4.30	3.85	258
	R inferior temporal	54	−54	−12	4.20	3.78	82
	SPL	0	−42	56	4.20	3.78	80
	R fusiform gyrus	38	−36	−26	4.17	3.76	45
SPECT	R PCC	4	−38	38	5.05	4.15	361
	R PGp (IPL)	44	−54	48	4.93	4.08	341
	L PGa (IPL)	−44	−60	32	3.85	3.38	163
	R PGa (IPL)	50	−58	28	4.15	3.58	95
	L inferior parietal	−36	−46	42	4.13	3.57	90

Voxel coordinates represent the peak voxel in local maxima, coordinates are expressed in Montreal Neurological Institute stereotactic space. *P* < 0.001, uncorrected. The regions with the extent under the expected voxels per cluster were omitted

*R* right, L left, *PCC* posterior cingulate cortex, *IPS* intraparietal sulcus, *IPL* inferior parietal lobule, *SPL* superior parietal lobule, *PGa* anterior angular gyrus, PGp: posterior angular gyrus

### Voxel-wise differences in CBF between PLD_1.5_ and PLD_2.5_

**Fig. 4:**
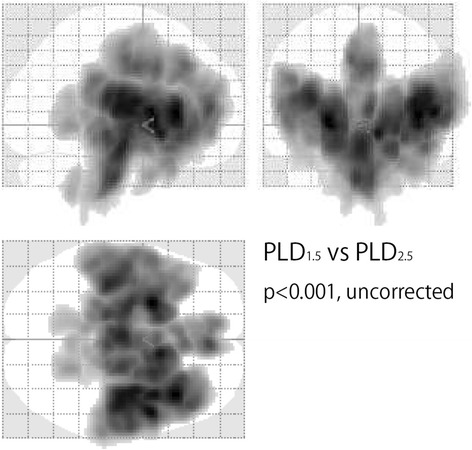
Voxel-wise CBF differences between PLD_1.5_ and PLD_2.5_ by paired t-test. PLD_1.5_ showed significantly higher CBF than PLD_2.5_ in the proximal areas of vascular territories of the anterior, middle, and posterior cerebral arteries (*p* < 0.001, *T* = 3.32, uncorrected)

PLD_1.5_ showed significantly higher CBF than PLD_2.5_ at the proximal areas of vascular territories of the anterior, middle, and posterior cerebral arteries (Fig 4).

## Discussion

From the results of our study, we identified significant positive correlations between CBF and cognition, when measuring using both ASL-MRI and SPECT. The main areas with significant results were located in PCC and temporo-parietal association cortexes, which are known to show decreases in perfusion or metabolism during cognitive decline in association with AD [25–28]. These areas are the posterior parts of the default mode network (DMN), which has a primary network center in the PCC. These regions have a strong functional connection to the left and right inferior parietal lobule (IPL), ventral and dorsal medial prefrontal cortex, and lateral temporal lobes [29]. Functional MRI studies have also consistently implicated the DMN as the most vulnerable network in AD [30, 31]. The posterior (temporo-parietal-predominant) DMN may be particularly susceptible in early-stage AD [32–34]. These studies therefore support our findings that a significant correlation exists between CBF and cognition, in the PCC and IPL. To the best of our knowledge, ours is the first report that succeeded in demonstrating such correlations between CBF and cognition using ASL-MRI, with supporting data using SPECT. These correlations may support the idea that regional CBF can serve as a biomarker of the neural changes underlying cognitive decline. As shown in Figs. 1b and 2b, the voxel values of SPM results dropped linearly with decreasing MMSE scores. Our results also showed similar significant correlations between cognition and CBF using ASL-MRI with PLD_1.5_ and PLD_2.5_, and SPECT. The measurement of CBF is thought to be influenced by modalities, tracers, and parameters. In fact, our results showed that ASL-MRI with PLD_1.5_ and PLD_2.5_ have significant differences. A significantly higher CBF was found at the adjacent areas of anterior cerebral arteries, middle cerebral arteries and posterior cerebral arteries for PLD_1.5_ compared to PLD_2.5_, suggesting early perfused areas. Liu et al. [35] evaluated the CBF of AD patients using ASL-MRI with PLD_1.5_ and PLD_2.5_, and identified lower CBF for both PLD durations at the specific area of AD pathology when compared to healthy control subjects, but with smaller clusters of voxel for PLD_2.5_. Despite of these significant differences in measured CBF using ASL-MRI with PLD_1.5_ and PLD_2.5_, PCC and temporo-parietal association cortexes were detected with significant correlations with cognition. This may suggest a possibility of the usefulness for the individual diagnosis using voxel-wise analyses of ASL-MRI. In Japan, voxel-wise analyses of SPECT using 3-Dimensional stereotactic surface projections (3D–SSP) [36] and an SPM-based method termed “easy Z-score imaging system (eZIS)” [37, 38] have been commonly used for the individual diagnoses in daily practices. Such voxel-wise methods may be helpful for making individual diagnoses using ASL-MRI. However, an age-specific normal database is required for the detection of significant abnormalities of individual images.

Our results also demonstrate a significant correlation between CBF and cognition at the right rectal gyrus for PLD_1.5_, and the right inferior temporal lobule and fusiform gyrus for PLD_2.5_. However, these areas are located at the edge of the brain, and these results might be caused by the errors during anatomical standardization and/or masking, and seem to be artifacts.

There were several limitations in this study. First, the sample size was limited; a larger number of subjects would be expected to provide more accurate results with correction for confounding variables, such as age and gender. Second, no images from cognitively normal subjects were used as controls, therefore we could not evaluate differences between AD patients and normal subjects. Third, the MNI template used for spatial normalization provided by SPM12 is based on a group of young, healthy individuals. The applicability of this template to older subjects is questionable. Fourth, the labeling efficiency plays a major role in PCASL quantification, but we did not calculate this parameter in this study. Furthermore, no conclusions could be drawn on the individual subject level, as the analyses were all performed on pooled data from each group.

## Conclusions

Significant positive correlations between CBF and cognition were detected using ASL-MRI with PLD_1.5_ and PLD_2.5_ in the PCC and temporo-parietal association cortexes. These results were supported by brain perfusion SPECT. CBF in these areas decreased with increasing severity of cognitive impairment. These measures could therefore be important in the clinical diagnosis and evaluation of AD. The difference of PLD made significant differences in the measured CBF, but not in the voxel-wise correlation with cognition.
